# Nutritional Approach to Small Intestinal Bacterial Overgrowth: A Narrative Review

**DOI:** 10.3390/nu17091410

**Published:** 2025-04-23

**Authors:** Sol Velasco-Aburto, Arancha Llama-Palacios, María Carmen Sánchez, María José Ciudad, Luis Collado

**Affiliations:** 1 Department of Medicine, Faculty of Medicine, University Complutense, 28040 Madrid, Spain; sol@solvelasconutricion.com (S.V.-A.); mallamap@ucm.es (A.L.-P.); mariasan@ucm.es (M.C.S.); 2 GINTRAMIS Research Group (Translational Research Group on Microbiota and Health), Faculty of Medicine, University Complutense, 28040 Madrid, Spain

**Keywords:** small intestinal bacterial overgrowth, SIBO, small intestine, low fermentation diet, intestinal dysbiosis

## Abstract

Small intestinal bacterial overgrowth (SIBO) is a functional digestive disorder whose incidence has been acknowledged by several medical associations, such as the American Gastroenterological Association. It is estimated that between 14% and 40% of patients diagnosed with irritable bowel syndrome also have SIBO, highlighting the importance of accurate diagnosis to enable effective treatment plans. Nutrition and diet therapy play a pivotal role in SIBO management, not only in alleviating symptoms but also in preventing relapses. The objective of this review is to gather updated information on dietary management for SIBO to define the role of the dietitian and determine the most suitable nutritional therapy based on scientific evidence. The review will encompass various strategies, ranging from specific diets to dietary supplements, as well as the potential contribution of dietary treatment to improving SIBO.

## 1. Introduction

Gut dysbiosis refers to any proliferation or change in the composition of the microbiota [1]. Small intestinal bacterial overgrowth (SIBO) is a type of gut dysbiosis that has recently garnered public attention despite its longstanding presence in the medical digestive field. As a result, some of the most important medical societies in the field, such as the American Gastroenterological Association [2] and the American College of Gastroenterology [3], along with the Asia Pacific consensus guideline [4], have taken a stance on the management of this disease. Today, thanks to the work of these medical associations and consensus guidelines, SIBO is recognized as a functional digestive disorder resulting from various pathologies and is more prevalent than previously thought [5]. It is estimated that up to 38% of patients diagnosed with irritable bowel syndrome (IBS) also have SIBO. Highlighting the need for accurate diagnoses to ensure adequate treatment [6]. Therefore, in patients with IBS, treatment of a positive SIBO test should be reserved for cases that have proven refractory to other diagnostic assessments.

Advances in metagenomics have facilitated a better understanding of SIBO, emphasizing its role in the broader context of intestinal microbiology [7]. Leading to novel management strategies, such as the use of probiotics and nutritional therapy.

However, SIBO relapses are common, occurring in up to 43% of patients within 9 months of antibiotic treatment [8]. Thus, addressing the underlying causes and improving lifestyle factors is important. In this sense, nutrition can play a key role. Prescribing a specific diet for the pathology can be advantageous for patients in terms of symptom control and preventing relapses [3,9]. This review will argue that dietitians can play a relevant role in the management of the disease by determining the most appropriate dietary therapy for SIBO. To do so, they must be familiar with the most recent and relevant information regarding the dietary management of SIBO. This familiarity will ensure that they can provide adequate and sufficient nutrition, avoid possible nutritional deficiencies, and prevent a worsening of the condition due to the implementation of inappropriate diets.

## 2. Small Intestinal Bacterial Overgrowth (SIBO)

SIBO is characterized by the presence of an excessive number of bacteria in the small intestine, leading to gastrointestinal symptoms [3]. These symptoms can be nonspecific and can overlap with other digestive disorders, given that they primarily involve abdominal distension, diarrhea, constipation, flatulence, indigestion, belching, and changes in gastrointestinal transit [10]. These symptoms are essentially due to the activity of gastrointestinal bacteria when they come into contact with fermentable carbohydrates in the diet [7]. SIBO can also produce extraintestinal symptoms, including asthenia or headache, as well as cardiovascular, endocrine, neurological, nephrological, connective tissue, or dermatological complications [11]. SIBO has been reported to be associated with an increase in lipopolysaccharides and inflammatory cytokines in the intestinal mucosa, which can cause intestinal hyperpermeability and contribute to chronic low-grade systemic inflammation [5].

In normal physiological situations and in sections of the digestive tract such as the colon, this bacterial activity supports the host’s digestive processes and has a myriad of beneficial functions [12]. However, this is not the case when these bacteria are present in excess, particularly in the small intestine.

Moreover, this excessive presence of bacteria in the small intestine can have additional negative consequences beyond the symptoms, such as malabsorption of certain nutrients like vitamin B12, steatorrhea, and deficiencies in fat-soluble vitamins [5]. These issues stem from interactions between the bacteria and their metabolites with these nutrients and have the potential to damage the intestinal mucosa [2].

**Figure 1:**
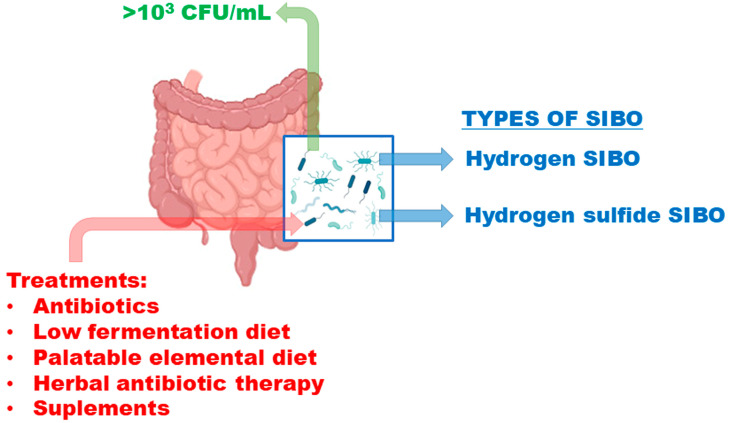
Types of SIBO and their possible treatments.

Additionally, depending on the type of bacteria that overgrow, the symptoms will vary slightly, as will the dietary and pharmacological treatment. Therefore, we identify 2 different types of SIBO depending on the gas produced by the various bacteria: (i) hydrogen SIBO, which typically presents with diarrhea, and (ii) hydrogen sulfide SIBO, which also generally presents with diarrhea [3] (Figure 1):

Another type of microbial overgrowth disorder involves the production of methane, where hydrogen is used as a substrate. In this case, the methanogens are archaea, not bacteria, and the overgrowth can occur in both the small and large intestines. Therefore, we refer to this condition as intestinal methanogen overgrowth (IMO), which is typically associated with constipation [3].

It is important to note that to date, the microbiota of the small intestine has not been precisely determined or defined, which impedes the categorization of bacterial populations as normal or abnormal [13]. Nonetheless, some initial studies characterizing the small intestine’s microbiota have suggested that gastrointestinal disorders might be less attributable to the quantity of bacteria and more to the type of bacteria present [13]. In the context of SIBO, certain bacterial populations, designated as disruptive, have been identified due to their propensity to displace other bacteria. Examples include *Klebsiella*, *Escherichia*, *Enterococcus*, and *Clostridium* [14]. Furthermore, a study that analyzed the bacterial composition of the mucosal samples of the duodenum, ileum, and sigmoid colon in individuals with and without SIBO found four genera (Lactobacillus, Prevotella_1, Dialister, and norank_f_Ruminococcaceae) were decreased in the individuals with SIBO compared with those without it [15].

Currently, the gold standard for determining the bacterial load in the small intestine relies on duodenal aspirates. Scientific consensus holds that normal bacterial levels in healthy individuals typically do not exceed 10^3^ colony-forming units (CFU) per ml. Therefore, an overgrowth of bacteria is defined as the presence of more than 10^3^ CFU/mL [16].

Although the threshold quantity defining pathological bacterial populations in the small intestine has been established, there is no equivalent consensus on the types of bacteria. This means that although an excessive quantity is detrimental to the patient, a clear definition of the normal microbiome of the small intestine is lacking. Therefore, it is difficult to determine whether specific types of bacteria indicate a pathological state of the intestinal microbiota in this section. Conversely, we also cannot definitively identify which bacteria are normal or beneficial and, therefore, desirable [13].

### 2.1. Causes of Small Intestinal Bacterial Overgrowth

Various factors have been associated with the onset of SIBO and could explain the higher prevalence in patients with these characteristics. The intestine has mechanisms that, if functioning properly, prevent the excessive proliferation of bacteria in the small intestine. These mechanisms include gastric acid, bile, and pancreatic enzymes, which have bacteriostatic and bactericidal properties, as well as peristaltic movements that propel food and bacteria toward the end of the digestive tract and the ileocecal valve, which prevents bacteria from the colon from migrating back into the small intestine [17].

However, if these mechanisms fail, the likelihood of developing SIBO increases. Several factors can render these mechanisms less effective. First, it has been demonstrated that SIBO is more prevalent in women and elderly individuals [10]. Additionally, its occurrence is common alongside other digestive disorders such as dyspepsia, intestinal motility dysfunction, and systemic sclerosis. Regarding other potential etiologies, SIBO has been associated with various conditions such as hypothyroidism, diabetes, pancreatitis, rosacea, Parkinson’s disease, and coronary heart disease, as well as with patients who have undergone abdominal surgeries [10].

A systematic review with meta-analysis has found an association between *Helicobacter pylori* and SIBO, particularly in younger adults [18]. A possible mechanism underlying this association involves the decrease in gastric acid concentration caused by atrophic gastritis due to *H. pylori* infection, which reduces the bactericidal effect of the stomach and allows bacteria to overgrow in the small intestine [19].

The use of certain medications is also linked to the development of SIBO, including opioids, which reduce intestinal motility. Moreover, there is a significant correlation between the continuous use of proton pump inhibitors and the subsequent prevalence of SIBO in these patients [20]; they inhibit gastric acid production, creating a less acidic environment in the stomach, which is thought to facilitate bacteria reaching the intestine more easily [20].

In the area of nutrition, studies have examined the impact of nutritional status and dietary habits on the development and prevalence of SIBO. A study on 60 patients with obesity and 60 controls found that those with obesity consumed more carbohydrates, more sugar, and less dietary fiber than the controls. The prevalence of SIBO in these individuals was 23.3%, compared with 6.6% in the patients with a normal body mass index (BMI), who consumed more fiber and fewer carbohydrates according to dietary surveys [21]. However, a limitation of this study is that although the results regarding the patients’ dietary patterns are intriguing, it does not clarify whether SIBO is a consequence of diet or obesity. Nor does it consider whether obesity could be a result of microbiota alterations, such as dysbiosis or SIBO itself. In this regard, other studies have attempted to relate body composition and anthropometric parameters to the production of specific gases and thus, specific subtypes of SIBO. One of them found that body composition parameters such as body weight, BMI, body fat, and mineral bone content might be inversely correlated with hydrogen production in patients with SIBO [22].

Other interesting studies have focused on personality and psychological determinants of SIBO. One found that higher levels of situational anxiety, stress, and neuroticism are more prevalent in patients with SIBO [23]. In another study, selected metabolites of the tryptophan kynurenine pathway were evaluated in depressive patients with SIBO. A higher level of kynurenine and quinolinic acid was found in patients with SIBO compared with the control group, suggesting that SIBO could be related to mental disorders [24].

### 2.2. Diagnosis of Small Intestinal Bacterial Overgrowth

As mentioned above, the gold standard for diagnosing bacterial overgrowth is the duodenal aspirate with a threshold of 10^3^ CFU/mL, above which the result is considered positive. However, this procedure is quite invasive for the patient, expensive, and carries the risk of sample contamination by the oral, gastric, and esophageal microbiota [25]. Therefore, the breath test is currently the most commonly used technique.

To assess microbial overgrowth, these tests use two possible substrates: glucose or lactulose. These substrates are fermented by the enteric microflora, producing gases that can subsequently be measured: methane and/or hydrogen [26]. There is no clear consensus as to which substrate is preferable. Glucose is a monosaccharide that is absorbed in the proximal intestine, whereas lactulose is a non-absorbable disaccharide that reaches the colon. Consequently, the use of glucose might increase the risk of false negatives since it could be absorbed in the small intestine before reaching the more distal segments where the bacteria could reside [27]. In contrast, the use of lactulose could result in another gas peak upon reaching the colon due to fermentation by the colonic microbiota. A test duration of 120 min is considered sufficient, given that the substrate is expected to transit through the small intestine within a maximum of 90 min [27].

Regarding diagnostic criteria, it has been determined that an increase of ≥20 ppm of hydrogen from baseline level within the first 90 min is considered positive for hydrogen SIBO. Additionally, a methane level of ≥10 ppm at any point during the test is indicative of IMO, as these patients tend to have elevated methane levels from the beginning of the test [28].

Furthermore, the urinary excretion tests using chemically synthesized bile acid conjugates have been used to diagnose SIBO. Although the number of clinical trials using these urinary excretion tests is small, the results have demonstrated the usefulness of bile acid conjugates as SIBO diagnostic substrates [29].

## 3. Treatment

Pharmacological treatment for SIBO has traditionally been empiric in patients with compatible symptoms. However, there is increasing recognition of the need to accurately diagnose not only the presence of SIBO but also the specific type of SIBO before prescribing antibiotic therapy [3]. The most common practice for treating hydrogen SIBO is the use of rifaximin, although other antibiotics such as metronidazole have also proven effective [28]. For IMO with overgrowth of methanogens, the combination of rifaximin and neomycin has yielded the best results [25]. A systematic review and meta-analysis found that patients with SIBO were 2.46 times more likely to experience symptomatic improvement when treated with antibiotics than when not treated with antibiotics [30]. The addition to standard antibiotic therapy of herbal supplements such as essential oils, berberine, or wormwood has also been studied with promising results [11].

It is important to highlight that recurrence after antibiotic treatment is quite common if the underlying causes of bacterial overgrowth are not resolved or if there are predisposing conditions such as older age, history of appendectomy, or chronic use of proton pump inhibitors [8]. Motility dysfunction appears to play a crucial role in the recurrence of SIBO after treatment, and thus, maintaining a healthy gut microbiome and adequate peristaltic movements should be the end goal of every SIBO treatment [31].

### 3.1. Dietary Management of Small Intestinal Bacterial Overgrowth

The nutritional repercussions of SIBO are numerous and should not be overlooked by healthcare professionals managing each case. Bacterial overgrowth in the small intestine can cause damage to the intestinal epithelium, it has metabolic implications due to bacterial activity, and it might reduce food intake in some patients due to fear of exacerbating symptoms. This can lead to consequences such as malabsorption, weight loss, and deficiencies in certain micronutrients [32]. Therefore, this review aims to highlight the importance of proper dietary counseling by a qualified dietician who understands the guidelines for managing this condition. The main dietary strategies that can be employed are discussed below.

### 3.2. Low Fermentation Diets

A diet low in fermentable oligosaccharides, disaccharides, monosaccharides, and polyols (FODMAP) is commonly recommended for the treatment of irritable bowel syndrome (IBS). However, it is increasingly popular for managing SIBO, with studies evaluating its potential to improve symptoms [9]. This is because the diet is based on eliminating foods that are rapidly fermentable, poorly absorbed in the small intestine, and osmotically active. Reducing their intake consequently decreases both osmotic activity and gas production, which, according to some studies, improves symptoms in patients with IBS and SIBO [9].

The group of fermentable carbohydrates in FODMAP includes oligosaccharides (fructans and galactans), disaccharides (lactose), monosaccharides (fructose), and polyols (sorbitol, mannitol, maltitol, xylitol, and isomaltose) [33].

Understanding how these carbohydrates are absorbed and digested in the body is important to understanding their effect on the gastrointestinal tract. First, fructose is absorbed in the small intestine by GLUT5 or GLUT2 transporters. Although GLUT5 can selectively transport fructose, GLUT2 is unable to transport fructose in the absence of glucose to co-transport it with [34]. This means that foods in which the ratio of glucose to fructose is not 1:1, and there is excess fructose, GLUT2 will not be able to fully absorb it [35]. Also, GLUT5 has a low transport capacity and is easily overloaded when there is excess fructose. This is easily achieved with products containing high fructose corn syrup, which is a major source of fructose in Western diets [36]. In either scenario, excess fructose continues through the gastrointestinal tract and reaches the colon, where it is fermented and causes symptoms, particularly in patients with IBS. However, when there is bacterial overgrowth in the small intestine, the symptoms are not the result of fructose malabsorption but rather fructose fermentation by excess bacteria [37]. In this case, foods with an excess fructose-to-glucose ratio will be more problematic due to the available fructose that they leave for bacterial fermentation. These foods include pears, apples, mangoes, and asparagus, among others [35].

Lactose, another FODMAP, should be hydrolyzed by lactase when it reaches the small intestine, and the resulting monosaccharides, glucose and galactose, are absorbed across the intestinal border via sodium glucose transporters [38]. Yet, in approximately 70% of the human population, lactase activity declines after the weaning phase, making most of the world’s population lactose intolerant [39]. In this case, lactose is not absorbed, and it increases the osmotic load, attracting water and causing diarrhea. It is then fermented by bacteria in the colon, producing short-chain fatty acids as well as gases such as hydrogen, methane, and carbon dioxide [40]. In patients with SIBO, excess bacteria ferment lactose in the small intestine before it can be fully absorbed, causing these typical lactose intolerance symptoms. In these patients, lactose breath tests can yield false positive results that are not due to a real intolerance but rather to bacterial overgrowth [41].

As for fructooligosaccharides and galactooligosaccharides, humans do not produce the enzymes required to hydrolyze them, so they are not absorbed and usually reach the colon, serving as prebiotics [42]. In cases of SIBO, they are fermented in the small intestine, followed by a symptomatic response.

Lastly, polyols are typically absorbed along the small intestine, with some of them reaching the colon and producing gas if not absorbed [35]. In the case of SIBO, they also serve as a substrate for fermentation in the small intestine.

The low-fermentation diets have three distinct phases. The first is characterized by FODMAP restriction; the second is for a gradual reintroduction to assess tolerance to each of the FODMAP groups; and the third is intended to be a personalized diet based on individual tolerance that can be maintained long-term [43].

For the first phase, high-FODMAP foods should be identified and restricted. The results are shown in the Table 1.

If the decision is made to prescribe this diet to patients with SIBO, a chart similar to the one presented here should be provided for the patient’s reference. Additionally, emphasis should be placed on the healthy foods that can and should be consumed to reduce the risk of patients adopting an inadequate diet that can lead to nutritional deficiencies. Therefore, it is recommended that gastrointestinal physicians work with a gastrointestinal dietitian who can personalize a balanced low-fermentation diet [43]. For patients with IMO, it is critical to ensure adequate fiber intake to prevent worsening constipation. Given that many high-fiber foods contain FODMAPs, designing a diet with sufficient fiber can be challenging. The easiest way to achieve this is to emphasize the importance of consuming low-fermentation fruits and vegetables such as citrus fruits, berries, leafy greens, root vegetables, zucchini, etc. It is also important to remember that although the low-fermentation diets are low in FODMAPs, they are not completely free of them. Symptoms are triggered by cumulative FODMAP consumption, so small amounts of these foods might still be tolerated [44].

After 4 to 6 weeks of restriction, the reintroduction phase should begin. In this phase, the patient, with the guidance of the dietitian, will be able to assess their tolerance to the various FODMAP groups. Many authors emphasize the importance of this phase because of the prebiotic nature of FODMAPs, the nutritional implication of long-term food restriction, and the difficulty of adhering to the diet when eating out [45].

Although the use of this diet is becoming more common for patients with both IBS and SIBO, some authors have highlighted the potential detrimental effects it can have on the gut microbiota if maintained long-term [9]. This is because, as stated, FODMAPs have prebiotic effects and modulate the microbiota by stimulating the growth of *Akkermansia muciniphila*, *Bifidobacterium*, and *Faecalibacterium prausnitzii*, as well as promoting the production of short-chain fatty acids [9]. Others state that the negative effects of excessive carbohydrate fermentation are larger than the possible imbalances caused by the diet [46]. In the treatment of intestinal pathologies such as SIBO, ensuring a state of intestinal eubiosis should be a therapeutic priority to prevent relapses and imbalances in the microbiota.

As noted, FODMAP implementation in practice is challenging, with limited long-term feasibility. A more practical approach may involve low-fermentation eating by eliminating common fermentable foods such as dairy, high-fructose items, and known gas-producing triggers like cauliflower, beans, Brussels sprouts, and sugar alcohols. Other elimination diets, like gluten-free or lactose-free, are also used by patients with SIBO. They may have a beneficial impact on gastrointestinal symptoms; however, they have adverse effects on the gut microbiota [47].

### 3.3. Elemental Diet

Elemental diets are easily digestible nutritional formulas that are almost entirely absorbed in the upper part of the gastrointestinal tract and contain the required daily allowance of vitamins, major/trace minerals, fat, free amino acids, and carbohydrates, but no fiber or fermentable carbohydrates of any kind [48]. Given their easily absorbable nutrients, their therapeutic benefits have been elucidated in several clinical settings, including Crohn’s disease (CD), chronic pancreatitis, and eosinophilic esophagitis/gastroenteritis (EoE/EGID) [49].

In the case of SIBO, their utility has been studied, with some research supporting their use due to their effects on the intestinal microbiota. These studies are based on the fact that when food is rapidly absorbed in the early sections of the small intestine, it deprives bacteria located further down of nutrients, thereby reducing their abundance. Although a 14-day regimen in 124 patients showed a positive effect in 80% of cases, with concomitant improvement in clinical symptoms, 11% dropped out of the trial due to an inability to tolerate the diet [50]. However, a prospective, open-label trial assessed the effect, tolerability, and safety of an exclusive two-week course of a novel palatable elemental diet (PED) in adult subjects with SIBO and/or IMO, where all the subjects completed the trial, and the PED significantly impacted the gut microbiome, including reductions in *Prevotella_9*, *Fusobacterium*, and *Methanobrevibacter smithii* [48].

## 4. Dietary Management After Treatment

As mentioned above, SIBO recurrence is not uncommon. Dietary changes to maintain remission in SIBO have not been rigorously studied, but data extrapolated from IBS trials suggests that there may be utility in a low-fermentation diet [25,51]. A low-fermentation diet theoretically could reduce the risk of recurrence as well. Low fermentation eating also incorporates meal spacing (at least 5 h between each meal) and avoidance of overnight eating, which further facilitates the occurrence of phase III migrating motor complexes in the small bowel [52]. Continuous grazing throughout the day may worsen bloating and related symptoms.

In addition, the primary cause of excessive fermentation in the small intestine is a malfunctioning gastrointestinal motor complex, which results in the gut’s longer retention of food residues [31]. Some food components have greater importance in the functioning of the gastrointestinal motor complex than others [31]. Moreover, some specific foods have a major impact on microbiota and gut motility, which are the main factors thought to influence the development of SIBO [31].

There is evidence that dietary fiber has beneficial effects on gut motility and can be used to prevent constipation [53]. Moreover, fruits high in fiber, sorbitol, and polyphenols, such as apricots and prunes, have been shown to improve gut motility [54]. On the other hand, foods rich in prebiotics are also vital to keep a diverse and healthy microbiome. Prebiotics are found in fruits, vegetables, grains, and other edible plants [55]. Other foods that could have a positive effect on gut motility and gut health include unsaturated fatty acids, specifically extra virgin olive oil [56], as well as foods high in polyphenols, which have been shown to simultaneously inhibit pathogens and stimulate beneficial bacteria [57]. Nevertheless, it is necessary to conduct research involving SIBO patients to confirm the role of prebiotics, unsaturated fatty acids, and polyphenols in the diet after treatment.

However, the Western diet, which is typically high in refined sugars, animal fats, processed meats, refined grains, salt, and other processed foods, and low in fruits, vegetables, whole grains, grass-fed animal products, fish, nuts, and seeds [58], has negative effects on overall health. In terms of gut health, it can cause dysbiosis, intestinal barrier dysfunction, increased intestinal permeability, and leakage of toxic bacterial metabolites into the circulation [58]. This can have many negative health consequences, as evidenced by studies linking sugar consumption to metabolic syndrome by promoting negative microbiota modulation and intestinal inflammation [59].

## 5. Dietary Supplements

### 5.1. Probiotics

Several studies have investigated the use of probiotics in the treatment of SIBO. Most interventions lasted from 2 to 4 weeks, with several cycles. Some were given as the main and only treatment, whereas others were combined with antibiotic therapy [60].

In one study comparing treatment with *Bifidobacterium* to a placebo, it was found that 81% of patients receiving the probiotic resolved SIBO compared with 25.4% in the placebo group. Additionally, the probiotic group reported a significant improvement in clinical symptoms [61].

Regarding the administration of probiotics along with antibiotic treatment, a study evaluated the effect of this combined approach. In this, patients with hydrogen SIBO were divided into three groups. One group received only the antibiotic metronidazole, the second group received only the probiotic *Saccharomyces boulardii*, and the third group received both the antibiotic and the probiotic. After 2 months, the group that combined the antibiotic and the probiotic had the highest eradication rate and the greatest reduction in gastrointestinal symptoms compared with the group that used only the antibiotic [62]. In the treatment of SIBO, *Saccharomyces boulardii* is preferred to bacterial probiotics because, as a yeast, it is unaffected when co-administered with antibiotics [63]. It is also more resistant to gastric acid and reaches the intestine in greater numbers [64].

In another study, the efficacy of probiotic therapy in alleviating SIBO and permeability in chronic liver disease was investigated. Six bacterial species (*Bifidobacterium bifidum*, *Bifidobacterium lactis*, *Bifidobacterium longum*, *Lactobacillus acidophilus*, *Lactobacillus rhamnosus*, and *Streptococcus thermophiles*) were used, and their administration in chronic liver disease was effective in alleviating SIBO and clinical symptoms but ineffective in improving intestinal permeability and liver function [65].

Furthermore, a study in which patients were supplemented for two weeks with the probiotic *Bifidobacterium infantis* 35624 (Align) could influence the outcomes of the lactulose breath test (LBT) for SIBO by significantly increasing methane excretion, while hydrogen levels remained unaffected [66]. The elevated methane concentrations reached thresholds typically considered positive for SIBO diagnosis. Consequently, the study recommended that patients discontinue probiotic use prior to undergoing LBT, as such supplementation may interfere with the accuracy of test results [66]. In addition, in a study designed to explore the association between brain fog and conditions such as SIBO, IMO, and other gastrointestinal disorders, it was found that over half of the patients with gastrointestinal conditions reported experiencing brain fog. Furthermore, these patients were significantly more likely to be using probiotics and proton pump inhibitors compared to those without symptoms of brain fog [67].

Currently, we can say that there is no probiotic treatment with sufficient evidence to recommend it to all patients [68]. Future studies should aim to determine which strains, in what context, and in what quantities probiotics might be beneficial for treating SIBO.

### 5.2. Herbal Antibiotic Therapy

With regard to the use of herbal antibiotics, several substances and preparations have been studied for their potential efficacy in reducing intestinal bacteria. Notable compounds include oregano and berberine [31]. One study tested the efficacy of herbal preparations, which are marketed in the United States under names such as Dysbiocide, FC Cidal, Candibactin-AR, and Candibactin-BR, showing promising results [69].

While some herbal therapies have been studied as alternatives to standard antibiotics, others have been studied as complementary treatment during and after standard antibiotic treatment [11].

Although there are indications that these types of compounds might be effective in treating SIBO, there is still insufficient evidence to determine the best treatment strategy with herbal antibiotics in these cases.

### 5.3. Other Supplements

Among other supplements that could be of interest in managing SIBO, partially hydrolyzed guar gum stands out. A 2010 study investigated the use of guar gum for the treatment of SIBO, with the aim of improving intestinal motility and assessing whether its use increased the effectiveness of antibiotic treatment with rifaximin. It was found that the consumption of 5 g of partially hydrolyzed guar gum along with the antibiotic was more effective than taking the antibiotic alone [70].

Also, a product containing xyloglucan and pea proteins was compared with simethicone for alleviating symptoms of functional abdominal bloating and distention and for reducing the hydrogen breath test used to diagnose SIBO. In this case, the product produced positive results, not only improving symptoms but also acting on SIBO etiology [71]. Xyloglucan has been shown to create a protective barrier on the intestinal mucus layer, reducing bacterial adherence and protecting tight junctions [72].

In light of this information, adding supplements to the treatment of SIBO might improve outcomes, and healthcare professionals treating these conditions should stay up to date to provide the most advanced and effective care.

## 6. Discussion

Although there is no evidence that a low-fermentation diet alone rebalances the microbiota of the small intestine or permanently reverses symptoms, it has been shown to significantly improve the patients’ symptoms over time. This diet relieves discomfort, abdominal distension, gas production, and diarrhea. Such symptomatic relief can help patients halt the weight loss caused by chronic diarrhea and, more importantly, improve their mood, energy levels, and quality of life. Patients with these symptoms often experience physical and psychological distress and can develop certain beliefs or fears about eating that are associated with symptoms worsening.

Therefore, along with antibiotic treatment, a low-fermentation diet is currently the most appropriate dietary therapy for these types of functional digestive disorders. However, as the studies mentioned in this review caution, this dietary strategy should be applied for a limited time because it reduces the availability of fermentable carbohydrates for the intestinal microbiota and might reduce its diversity, potentially causing more problems than benefits. Moreover, the low-fermentation diet, due to its reduced fiber intake, can worsen constipation and has a higher glycemic index than a high-fiber diet. These factors are important to consider for individuals who are prone to constipation and for those with conditions such as diabetes or prediabetes.

Lastly, it is crucial in these types of digestive disorders to make a proper differential diagnosis and rule out other conditions with overlapping symptoms, such as celiac disease, IBS, non-celiac gluten sensitivity, and inflammatory bowel disease.

In terms of diet therapy, individualization is paramount. Each patient can respond differently to the diet, tolerating foods they theoretically should not or not tolerating foods they should. Additionally, each patient’s habits, preferences, aversions, and socioeconomic status should be considered when designing a specific diet. The goal should always be to make the diet as non-restrictive as possible, reduce the risk of nutritional deficiencies, and, when possible, use nutrition education tools to promote healthier eating.

## Figures and Tables

**Table 1:** FODMAP groups and their corresponding foods. Based on the information from Zugasti Murillo et al. [33].

Group	Type	Present in	Foods to Avoid
Oligosaccharides	Fructans	Cereals, vegetables, legumes, fruits, and inulin	Bread, cookies, and breakfast cereals containing wheat. Artichokes, asparagus, onions, garlic, leeks, lentils, chickpeas
	Galactans	Legumes, cruciferous vegetables, and nuts	Beans, chickpeas, soybeans, Brussels sprouts, walnuts, cabbage
Disaccharides	Lactose	Milk and dairy products	Milk, yogurt, ice cream, custards, soft cheeses
Monosaccharides	Fructose	High fructose corn syrup, fruits, vegetables, and honey	Apples, cherries, mangoes, pears, watermelon, asparagus, artichokes, honey, and HFCS in processed foods, beverages, and nectars
Polyols	Sorbitol	Fruit	Apples, apricots, avocados, blackberries, cherries, nectarines, pears, plums, raisins
	Mannitol, Maltitol, Xylitol, Isomaltose	“Low-calorie” and “sugar-free” products	Candies, chewing gum, ice cream, cake

## Data Availability

Not applicable.

## Abbreviations

The following abbreviations are used in this manuscript:

SIBO	small intestinal bacterial overgrowth
IBS	irritable bowel syndrome
FODMAP	fermentable oligosaccharides, disaccharides, monosaccharides and polyols
BMI	body mass index
